# Unintended Pregnancy among HIV Positive Couples Receiving Integrated HIV Counseling, Testing, and Family Planning Services in Zambia

**DOI:** 10.1371/journal.pone.0075353

**Published:** 2013-09-30

**Authors:** Kristin M. Wall, Lisa Haddad, Bellington Vwalika, Naw Htee Khu, Ilene Brill, William Kilembe, Rob Stephenson, Elwyn Chomba, Cheswa Vwalika, Amanda Tichacek, Susan Allen

**Affiliations:** 1 Rwanda Zambia HIV Research Group, Department of Pathology & Laboratory Medicine, School of Medicine, Emory University, Atlanta, Georgia, United States of America; 2 Department of Epidemiology, Rollins School of Public Health, Emory University, Atlanta, Georgia, United States of America; 3 Department of Gynecology and Obstetrics, Emory University, School of Medicine, Atlanta, Georgia, United States of America; 4 Department of Gynecology and Obstetrics, School of Medicine, University of Zambia, Lusaka, Zambia; 5 Department of Epidemiology, Ryals School of Public Health, University of Alabama at Birmingham, Birmingham, Alabama, United States of America; 6 Hubert Department of Global Health, Rollins School of Public Health, Emory University, Atlanta, Georgia, United States of America; 7 Ministry of Community Development, Mother and Child Health, Lusaka, Zambia; Wits Reproductive Health and HIV Institute, South Africa

## Abstract

**Objective:**

We describe rates of unintended pregnancy among HIV positive couples in Lusaka, Zambia. We also identify factors associated with unintended pregnancy among oral contraceptive pill (OCP) using couples in this cohort.

**Design:**

Data were analyzed from couples randomized in a factorial design to two family planning intervention videos.

**Methods:**

Rates of unintended pregnancy were stratified by contraceptive method used at time of pregnancy. Predictors of time to unintended pregnancy among OCP users were determined via multivariate Cox modeling.

**Results:**

The highest rates of unintended pregnancy were observed among couples requesting condoms only (26.4/100CY) or OCPs (20.7/100CY); these rates were not significantly different. OCP users accounted for 37% of the couple-years (CY) observed and 87% of unintended pregnancies. Rates of unintended pregnancy for injectable (0.7/100CY) and intrauterine device (1.6/100CY) users were significantly lower relative to condom only users. No pregnancies occurred among contraceptive implant users or after tubal ligation. Factors associated (p<0.05) with time to unintended pregnancy among OCP users in multivariate analysis included the man wanting more children, the woman being HIV negative versus having stage IV HIV disease, and the woman reporting: younger age, no previous OCP use, missed OCPs, or sex without a condom.

**Conclusions:**

Long-acting reversible contraceptive methods were effective in the context of integrated couples HIV prevention and contraceptive services. Injectable methods were also effective in this context. Given the high user failure rate of OCPs, family planning efforts should promote longer-acting methods among OCP users wishing to avoid pregnancy. Where other methods are not available or acceptable, OCP adherence counseling is needed, especially among younger and new OCP users.

**Trial registration:**

ClinicalTrials.gov NCT00067522

## Introduction

Though the Total Fertility Rate (TFR), or average number of births a woman has in her lifetime, has declined in sub-Saharan Africa from 6.5 in 1950 to 5.1 in 2010, this trend has not been sufficient to address the region’s rapid population growth of 2.5% per year [1]. As fertility preferences are changing, family planning programs are falling short in meeting couples’ fertility needs. The Demographic and Health Surveys estimate that 10–65% of all pregnancies in the region are unintended, varying by country and age group [2]. Preventing unintended pregnancy via contraception, a key public health challenge, reduces maternal and child mortality, rates of abortion, and poverty while improving socioeconomic status, access to education, and gender equity [3]–[7]. Up to an estimated 40% reduction in maternal deaths related to unintended pregnancy could be achieved with contraceptive use [6]. Additionally, in countries with generalized HIV epidemics and low contraceptive use, improving family planning services among HIV positive and high-risk women is critically important to reduce unintended pregnancy and the burden of mother-to-child transmission of HIV.

Unintended pregnancies occur primarily due to unmet need for contraception as well as reliance on less effective, user-dependent, short-acting methods. Unmet contraceptive need in sub-Saharan Africa is high, surpassing 30% in some countries [8]. About one-third of unintended pregnancies occur among women accessing contraception, many of whom are using short-term methods that require user adherence on a daily or quarterly basis such as condoms, oral contraceptive pills (OCPs), and depo-medroxyprogesterone acetate (DMPA) injectables [9], [10]. Differential obstacles in adherence to short-term versus long-term reversible contraceptive methods, such as the intrauterine device (IUD) and implant, translate into differences in typical-use method failure rates. Estimated 1-year failure rates among women with typical-use are 15% for condoms, 8% for OCPs, 3% for DMPA, and <1% for the IUD and implant [11], [12].

Difficulties with contraception adherence are compounded in limited resource countries where economic and infrastructure barriers, such as transportation costs/reliability or clinic supply chain interruptions, are obstacles to the repeat clinic visits required for adherence to shorter-acting methods [13]–[16]. Published data from several studies in sub-Saharan Africa suggest much higher typical-use failure rates. Some HIV prevention studies, including phase 2 and 3 microbicide trials, have observed annual pregnancy rates of up to 64/100 PY despite monthly contraceptive counseling [17]–[19].

Without the need for repeat clinic visits or daily adherence, long-acting reversible contraceptive (LARC) methods provide 3–5 years of protection from pregnancy with contraceptive implants (Jadelle, Sino-Implant, Implanon) and up to 12 years with the copper IUD [20]–[22]. Additionally, these methods are more cost-effective over time. Median direct cost per couple-year of protection against unintended pregnancy is lowest for the IUD ($1.64) and Sino-implant ($4.02) and is similar for Jadelle, DMPA, and OCPs ($7.90 to $8.70) [23]. However, use of these highly effective and cost-effective LARC methods remains extremely low; use of modern methods among married Zambian women is 11% OCP, 8.5% injectable, 0.4% implant, and 0.1% IUD [24].

In Zambia, where HIV prevalence among reproductive age individuals is high (23.1% in urban areas and 10.8% in rural areas) [25], prevention of maternal-to-child HIV transmission and unintended pregnancy are dual priorities that could be addressed through integrating HIV prevention and family planning services [26]–[29]. Unfortunately these programs are typically organized vertically, and integration can be difficult. Women often cite the importance of male partners in contraceptive method choice, continuation, and consistent condom use [30]–[32], and HIV services that involve partners (such as couples’ voluntary HIV counseling and testing, CVCT), may observe higher typical-use contraception efficacy rates, particularly for the user-dependent methods [33].

We previously reported that a video-based intervention increased LARC method uptake among couples seeking CVCT in Lusaka, Zambia [34]; however, that intervention increased time to pregnancy only among HIV positive women who were already contracepting prior to the study [35]. The impact of contraceptive method choice on unintended pregnancy, among both HIV positive and high-risk HIV negative women in regions with high fertility and HIV incidence, remains largely unknown. The present analysis examines rates of unintended pregnancy among couples choosing different contraceptive options after the intervention, as well as predictors of unintended pregnancy among couples using OCPs, in order to improve program planning and patient counseling.

## Methods

### Ethics Statement

The study was approved by the Institutional Review Boards at Emory University and the University of Zambia. Written informed consent was obtained from all study participants.

### Study Design

The demographics of the cohort [36]; knowledge of, prior experience with, and concerns about modern contraceptive methods [37]; impact of informed consent on contraceptive knowledge [38] and planning behaviors [39]; study and intervention design and uptake of contraception immediately after the intervention [34]; and impact of the video-based interventions on incident pregnancy [35] have been previously reported.

Briefly, we analyzed data from 1060 couples recruited from CVCT services in Lusaka, Zambia. Eligible couples had no contraception contraindications and were cohabiting at least 12 months, fecund, not currently pregnant, and concordant HIV positive (man and woman both positive) or HIV discordant (man and woman have different results). Eligible men and women were ages 18–65 and 18–45, respectively. Participants were randomized in a factorial design to view video-based interventions. A “Methods” video detailed family planning methods with a focus on LARC methods, a “Motivational” video modeled future planning behaviors, and a “Control” video contained information on topics such as hand washing, bed-nets, and nutrition. After the intervention, couples were offered the full range of contraceptive methods on-site and were followed at 3-month intervals, with the possibility for interim visits. Condoms were provided at each visit to all couples. Couples in which the man wanted children within the next year were not included in this analysis as they were not considered to be at risk of adopting a method.

### Definitions

We defined a pregnancy as planned if it occurred after a woman discontinued her method because she reported a desire to become pregnant. All other pregnancies were considered unintended, specifically those occurring among women who reported using condoms and/or another contraceptive method and did not express a desire for pregnancy.

### Analysis

Pregnancy incidence rates (number of pregnancies over the total couple-time) among women who did not desire pregnancy were calculated and stratified by the contraception method being used when the pregnancy occurred. Couple-time was attributed a method only when the woman was an active user. Thus, couples could contribute follow-up time to multiple methods. Significant differences between the rates of unintended pregnancy for each method relative to couples using only condoms were calculated using Mid-p exact tests.

OCP using couples were described (n/mean, %/SD) by fixed covariates of interest (including trial arm, sociodemographic characteristics, family planning and behavioral factors, and HIV-related health characteristics), and stratified by whether they experienced a pregnancy. Significant differences in the distribution of these covariates by pregnancy status were determined with Chi-square (or Fisher’s Exact) tests.

Among OCP users only, we evaluated univariate associations between time to unintended pregnancy and fixed covariates as well as time-varying health and sexual-related covariates assessed at 3-monthly and interim visits via crude hazards ratios (cHRs). We then built a multivariate Cox model containing covariates significant (p<0.05) in the univariate. Multi-collinearity between model variables was assessed (taking condition indices >30 and variance decomposition proportions >0.5 as cutoffs). The proportional hazards assumption was confirmed for the time-independent covariates. Adjusted hazards ratios (aHRs) are presented for covariates in the final multivariate model. Data analysis was conducted with SAS v9.3 (Cary, NC).

## Results

### Unintended Pregnancy Rates

There were 137 pregnancies during 1428.7 couple-years (CY) of follow-up, 126 of which were unintended. The overall unintended pregnancy incidence rate was 8.8/100CY. Women who requested condoms only (26.4/100CY) and those using OCPs (20.7/100CY) experienced the highest rates of unintended pregnancy. These rates were not significantly different. The majority (87%) of all unintended pregnancies occurred among women using OCPs, who accounted for 37% of the CYs observed (Table 1).

**Table 1:** Unintended pregnancy incidence rates by method used at time of pregnancy.

	Number of unintended pregnancies	Follow-up time (CY)	Pregnancy incidence per (100 CY)	95%CI	p value*
**Pregnancies occurring on:**					
Condom§	12	45.42	26.42	14.31, 44.92	ref
Oral contraceptive pill	109	527.24	20.67	17.06, 24.84	0.415
Injectable#	4	541.07	0.74	0.23, 1.78	<0.0001
Intrauterine device ?	1	64.71	1.55	0.08, 7.62	<0.001
Implant	0	221.15	0.00	0.00, 8.51	<0.0001
Tubal ligation	0	29.09	0.00	0.00, 6.37	0.003
**Total**	**126**	**1428.68**	**8.82**	**7.38, 10.46**	

* 2-tail Mid-p exact test p value.

§ Chose to receive no method other than condoms, which were provided to all couples.

**#4 pregnancies on Injectable: N = 1 user failure** (22 weeks between injection and pregnancy date); **N = 2 user failures** (24 weeks between injections); **N = 1 neither method nor user failure** (Client likely in very early stages of pregnancy at enrollment, undetected by initial pregnancy test).

? 1 **pregnancy on Intrauterine device: N = 1 method failure** (Intrauterine device was protruding at external os of cervix and easily removed at the time pregnancy detected).

The pregnancy incidence among women using injectables was 0.7/100CY and among women using IUDs was 1.6/100CY, with no pregnancies occurring on the contraceptive implant or after tubal ligation. Pregnancy rates were significantly lower (p<0.05) for injectable, IUD, contraceptive implant, and tubal ligation users relative to condom-only users.

Three of the four pregnancies occurring on injectables were classified as user failures (i.e., women were non-adherent with >15 weeks between the last injection and pregnancy date). The remaining pregnancy was likely in very early gestation at enrollment, with the pregnancy only becoming manifest after an initial negative pregnancy test and after the first injection was given. One IUD expulsion (a method failure) resulted in a pregnancy (Table 1 footnotes).

### Descriptive Results: OCP Users

Five hundred and thirteen women used OCPs at some point during follow-up, of whom 109 (21.2%) experienced an unintended pregnancy. OCP users were on average 28.4 years of age for women and 34.4 years of age for men, and couples had an average of 2.1 living children at baseline. OCP users who did not become pregnant were older and had a higher monthly household income relative to those with a pregnancy (p<0.05).

Most women (63%) and men (52%) did not want more children. The man’s desire for a child was associated with significantly unintended pregnancy (p = 0.002); among couples experiencing an unintended pregnancy, 57% of men reported wanting a child, though not in the next year, at baseline versus 39% for non-pregnant couples.

Almost three-quarters (73%) of couples were using no modern method of contraception pre-randomization. About two-thirds of women (68%) reported ever having used OCPs previously. Significantly fewer women experiencing unintended pregnancy reported previous OCP use (57%) compared with those not experiencing an unintended pregnancy (71%) (p = 0.009). At enrollment, 27% of women had ever used injectables, and only 2% reported ever using implant or IUD. Worries, concerns, or fears about contraception reported by women were highest for OCPs (88%) and lowest for the implant and IUD (37% and 39%, respectively). Most (89%) women reported engaging in sex without a condom with the study partner at some point during follow-up, and 95% women reported having missed taking at least one OCP at some point during follow-up.

Over two-thirds (68%) of couples were concordant HIV positive. OCP using women who became pregnant during follow-up were less likely to have advanced (stage IV) HIV disease relative to OCP using women who did not experience a pregnancy (2% versus 13%) (Table 2).

**Table 2:** Fixed sociodemographic, family planning and behavioral, and health characteristics among OCP users.

	Total OCP users (N = 513)		Unintended pregnancy during follow-up (N = 109)		No pregnancy during follow-up (N = 404)		
	n/mean	%/SD	n/mean	%/SD	n/mean	%/SD	p value*
**Arm of trial**							
Methods video	240	47%	51	47%	189	47%	0.999
No Methods video	273	53%	58	53%	215	53%	
**Arm of trial**							
Motivational video	259	50%	48	44%	211	52%	0.129
No Motivational video	254	50%	61	56%	193	48%	
***Sociodemographics***							
**Age of woman**	28.43	5.49	26.46	5.29	28.96	5.43	<0.0001
**Age of man**	34.38	6.13	33.07	6.35	34.74	6.03	0.015
**No. living children**	2.08	1.36	2.05	1.38	2.09	1.36	0.788
**Monthly household income (USD)**	67.12	112.58	49.02	48.28	71.96	123.86	0.003
***Family planning and behavioral***							
**Woman fertility intentions**							0.146
Wants more children in the next year	30	6%	7	6%	23	6%	
Wants more children, not in next year	148	29%	40	37%	108	27%	
Does not know	11	2%	3	3%	8	2%	
Does not want more children	323	63%	58	54%	265	66%	
**Man fertility intentions**							0.002
Wants more children, not in next year	219	43%	62	57%	157	39%	
Does not know	27	5%	6	6%	21	5%	
Does not want more children	266	52%	40	37%	226	56%	
**Contraception methods ever used (past or at enrollment)**							
OCPs	345	68%	62	57%	283	71%	0.009
INJ	135	27%	32	30%	103	26%	0.416
IMP or IUD	11	2%	3	3%	8	2%	0.849
**Woman has worries, concerns, or fears about:**							
OCPs	449	88%	93	85%	356	89%	0.260
INJ	341	67%	73	68%	268	67%	0.856
IMP	167	37%	28	31%	139	39%	0.145
IUD	176	39%	36	37%	140	40%	0.613
BTL	171	43%	27	34%	144	45%	0.090
**Sex with partner in study without a condom reported by women (ever during follow-up)**							0.803
Yes	455	89%	96	88%	359	89%	
No	58	11%	13	12%	45	11%	
**Missed OCPs (ever during follow-up)**							
No missed OCPs reported	27	5%	2	2%	25	6%	0.071
Any missed OCPs reported	486	95%	107	98%	379	94%	
***Health: HIV***							
**HIV serostatus at enrollment**							0.241
Serodiscordant (woman is positive)	81	16%	22	20%	59	15%	
Serodiscordant (man is positive)	84	16%	20	18%	64	16%	
Concordant positive	348	68%	67	61%	281	70%	
**Stage of HIV of woman**							0.004
HIV negative	84	16%	20	18%	64	16%	
Stage I–II	216	42%	56	51%	160	40%	
Stage III	159	31%	31	28%	128	32%	
Stage IV	54	11%	2	2%	52	13%	
**Stage of HIV of man**							0.440
HIV negative	81	16%	22	20%	59	15%	
Stage I–II	194	38%	42	39%	152	38%	
Stage III	184	36%	36	33%	148	37%	
Stage IV	54	11%	9	8%	45	11%	

* 2-sided Chi-square (or Fisher’s Exact) test p value.

OCP: oral contraceptive pill; INJ: injectable contraception; IUD: intrauterine device; IMP: implant; BTL: bilateral tubal ligation; USD: United States Dollar.

Numbers may not add to column totals due to missing values or if question inapplicable.

Additional factors evaluated and not found to be significant included: Woman understands Nyanja, man understands Nyanja, who decides when/if you should have children (reported by woman), who decides when/if you should have children (reported by man), number of lifetime sexual partners reported by woman (per partner increase), age at first intercourse reported by woman (per year increase), and the following time varying health factors: heavy menstrual bleeding, irregular bleeding, dyspareunia, lower abdominal pain, bleeding between periods, cystitis/dysuria, vaginal discharge, acute genital ulcer.

### Modeling Results: OCP Users

Woman’s and man’s age were collinear and only woman’s age was retained in the final model (Table 3). In a multivariate Cox model containing time-varying covariates, factors associated (p<0.05) with unintended pregnancy among OCP users were the younger age of the woman partner (aHR = 0.95 per year increase), the man wanting more children (though not in the next year) at baseline (aHR = 1.58), the woman never having used OCPs previously (aHR = 1.54), the woman reporting any sex without a condom (aHR = 1.83), and the woman reporting any missed OCPs (aHR = 1.86). Women with Stage IV HIV disease at baseline were less likely to become pregnant during follow-up (aHR = 0.16). None of the time-varying health characteristics were significant (p<0.05) in the multivariate model. The “Methods” and “Motivational” intervention videos did not impact the time to unintended pregnancy among OCP users, nor did the interaction of both videos.

**Table 3:** Extended Cox models of factors associated with time to unintended pregnancy among OCP users.

	UNIVARIATE				MULTIVARIATE			
	cHR	95%CI		p value	aHR	95%CI		p value
**Arm of trial**								
Methods video	ref							
No Methods video	1.03	0.71	1.50	0.884				
**Arm of trial**								
Motivational video	ref							
No Motivational video	1.13	0.79	1.61	0.516				
***Sociodemographics***								
**Age of woman (per year increase)**	0.92	0.88	0.96	<.0001	0.95	0.91	0.99	0.014
**Age of man (per year increase)**	0.95	0.92	0.99	0.005	n/a			
**No. living children (per child increase)**	0.96	0.79	1.16	0.670				
**Monthly household income (per 2 USD increase)**	0.99	0.99	1.00	0.069				
***Family planning and behavioral***								
**Woman fertility intentions**								
Wants more children in the next year	1.17	0.53	2.57	0.701	1.00	0.45	2.22	0.999
Wants more children, not in next year	1.65	1.10	2.47	0.016	1.25	0.82	1.92	0.308
Does not know	1.06	0.33	3.39	0.922	0.87	0.27	2.83	0.812
Does not want more children	ref				ref			
**Man fertility intentions**								
Wants more children, not in next year	1.85	1.24	2.76	0.003	1.58	1.03	2.44	0.038
Does not know	1.47	0.62	3.48	0.377	1.40	0.58	3.37	0.454
Does not want more children	ref				ref			
**Contraception methods ever used (past or at enrollment) (ref = Yes)**								
OCPs	1.58	1.08	2.31	0.019	1.54	1.01	2.35	0.043
INJ	0.93	0.61	1.40	0.719				
IMP or IUD	0.47	0.15	1.47	0.194				
**Woman has worries, concerns, or fears about (ref = No)**								
OCPs	0.74	0.43	1.25	0.260				
INJ	0.97	0.65	1.46	0.897				
IMP	0.82	0.53	1.29	0.397				
IUD	0.96	0.64	1.45	0.846				
BTL	0.75	0.47	1.20	0.228				
**Sex with partner in study in past 3 months without a condom reported by woman (time-varying), if any sex reported**								
No times	ref							
≥1 time	1.77	1.20	2.61	0.004	1.83	1.23	2.72	0.004
**Missed OCPs (in last 3 months, time-varying)**								
No missed OCPs reported	ref							
Any missed OCPs reported	2.38	1.56	3.64	<.0001	1.86	1.18	2.91	0.007
***Health: HIV***								
**HIV serostatus at enrollment**								
Serodiscordant (woman is positive)	1.40	0.86	2.27	0.172				
Serodiscordant (man is positive)	1.32	0.80	2.17	0.283				
Concordant positive	ref							
**Stage of HIV of woman**								
HIV negative	ref				ref			
Stage I–II	1.05	0.63	1.74	0.866	0.80	0.46	1.37	0.411
Stage III	0.74	0.42	1.30	0.289	0.62	0.34	1.15	0.128
Stage IV	0.15	0.04	0.66	0.012	0.16	0.04	0.68	0.014
**Stage of HIV of man**								
HIV negative	ref							
Stage I–II	0.80	0.48	1.34	0.390				
Stage III	0.74	0.44	1.26	0.271				
Stage IV	0.65	0.30	1.41	0.272				

OCP: oral contraceptive pill; INJ: injectable contraception; IUD: intrauterine device; IMP: implant; BTL: bilateral tubal ligation; USD: United States Dollar; cHR: crude Hazard Ratio; aHR: adjusted Hazard Ratio.

Additional factors evaluated and not found to be significant in the univariate analysis included: Woman understands Nyanja, man understands Nyanja, who decides when/if you should have children (reported by woman), who decides when/if you should have children (reported by man), number of lifetime sexual partners reported by woman (per partner increase), age at first intercourse reported by woman (per year increase), Method use pattern (Always continued initial method selected versus switched methods at least once during followup), and the following time varying factors: heavy menstrual bleeding, irregular bleeding, dyspareunia, lower abdominal pain, bleeding between periods, cystitis/dysuria, vaginal discharge, acute genital ulcer**.**

## Discussion

We present results from a prospective cohort of HIV discordant and concordant positive couples receiving CVCT, a video-based intervention to promote family planning, and access to the full range of contraceptive options. The overall pregnancy rate was 8.8/100CY, and ranged from 21–26/100CY in those using OCPs or condoms only, respectively, to 0.0–1.6/100CY among couples using injectables, LARC, or permanent methods. Remarkably, less than 10% of pregnancies were planned. Of unintended pregnancies, 87% occurred among OCP users who almost universally reported sexual exposures without a condom and missed pill doses. As these women only represented 37% of the couple-years observed, OCPs are not only the highest-risk non-barrier method, but OCP users comprised our largest risk group. For this reason, we focused our analysis on unintended pregnancies occurring among OCP users.

Women using injectables in our cohort also experienced low rates of unintended pregnancy (0.7/100CY), similar to LARC users. This is in marked contrast to other published findings of typical-use failure rates for injectables, which are higher relative to the IUD and implant. For example, Ngure et al (2012) report pregnancy rates of 6–7/100CY for injectable using HIV serodiscordant couples from seven African countries, with no pregnancies occurring on LARC methods [40]. The high adherence to injectables we observed may be in part due to integrated family planning and HIV prevention services that included regular study visits and male partner involvement. Additionally, couples enrolled in the study were provided reimbursement for transportation thus eliminating one potential barrier for the repeat clinic visits needed for injectable adherence.

Predictors of unintended pregnancy among OCP users from our modeling analysis can target interventions to this high-risk group. Consistent with other studies in the region, we found that the younger age of the woman, the man wanting more children, and the woman reporting sex without a condom were associated with unintended pregnancy among OCP users [35], [40]–[42]. In contrast with other studies, we did not find that the number of prior pregnancies was associated with unintended pregnancy.

The high prevalence of unprotected sex and its association with unintended pregnancy among OCP users underscores the challenges of combining barrier methods with effective contraception in our cohort of HIV concordant positive and discordant couples. Improved fertility based HIV prevention counseling that addresses dual method use is critical for high-risk couples. Though our limited sample size of discordant couples precluded evaluation of differences in unprotected sex across contraceptive types, we have previously published findings from Rwanda and Zambia showing that discordant couples who selected LARC methods reported significantly less unprotected sex relative to couples using condoms only [43]. Additionally, although we did not find that fears or concerns about contraception were related to unintended pregnancy among OCP users, the substantial proportion of couples that expressed concern regarding contraceptive method safety highlights an important challenge for educational and social marketing programs.

Women who were new to OCP use were at higher risk of unintended pregnancy relative to women who had previous experience with the method. The ways in which women who are naïve to a method differ from experienced users should be investigated further as adherence counseling may need reinforcement with new users. Additionally, reporting any missed OCPs in the prior three months was associated with unintended pregnancy. Self-reported adherence to OCPs is challenging to evaluate and often inaccurate. Women who become pregnant may be more likely to report missed pills, a potential recall bias [44], [45]. Given that the majority of couples wished to cease or limit childbearing, promotion of LARC uptake among OCP users is indicated.

We observed that advanced HIV stage of the woman was associated with decreased unintended pregnancy incidence relative to being HIV negative. In prior studies in Rwanda, we similarly observed that the greatest reduction in incident pregnancy, after receipt of information on and access to hormonal contraception, was observed for HIV positive women [28], [46]. However, the relationships between HIV positive serostatus, advanced HIV disease progression, fertility intentions, and pregnancy outcomes are complex. For example, HIV positive serostatus awareness and associated changes in fertility intentions do not always translate into corresponding changes contraception use, as observed among HIV positive and negative couples in northern Malawi [47], and nuanced gender-specific health concerns and fertility intentions have been observed in Malawi after an HIV diagnosis [48]. In our study, health concerns related to advanced HIV disease may have influenced desires to avoid pregnancy, leading to more vigilant contraceptive use or reduced coital frequency, or chronic disease progression may have led to reduced coital frequency or fecundity.

It is notable that men’s but not women’s fertility intentions were predictive of unintended pregnancy among OCP users. This finding highlights the importance of male partner involvement and assessing fertility intentions of the couple. Considerable gender discordance between health and contraception knowledge, sexual agreements, and fertility intentions has been noted previously [37], [49], [50] and can be addressed by involving both members of the couple in joint family planning counseling.

In this analysis among OCP-using couples, the family planning intervention videos were not predictive of unintended pregnancy. Though exposure to the one-time video intervention influences initial method choice [34], it may not be enough for long-term impact, the effect overshadowed by subsequent method discontinuation or non-adherence. Repeated messaging may be required to promote method continuation and adherence.

We cannot determine from our data the potential impact of drug interactions on contraceptive failures. As indicted in the WHO Medical Eligibility Criteria, there is evidence that various drugs may reduce the efficacy of some combined hormonal contraceptives, and it is recommended to avoid these drug combinations in clinical practice. In populations where these drugs are prevalent, this would lend further support to promoting other contraceptive choices with no drug interactions, such as the IUD.

Strengths of this study include its prospective design and measurement of important time-varying health characteristics. Couples were able to select their methods of contraception thus making our findings more generalizable to a practical setting than if methods had been randomized. Additionally, in contrast to studies which use retrospective measures of unintended pregnancy, our study does not suffer from potential post-birth rationalization bias which can lead to underestimates of unintended pregnancy [1]. We believe assessing pregnancy intentions at the time a contraceptive method is initiated or discontinued to be a more accurate measure.

Limitations of our study include the restricted external generalizability of our findings outside of HIV prevention contexts, and a possible self-selection bias for couples with more motivation or interest in family planning who may exhibit improved overall adherence behaviors. We did not use the Bonferroni correction for multiple comparisons as little is known about predictors of unintended pregnancy and we thought this method too conservative to detect potentially weak signals – we acknowledge therefore that our potential for type II error is higher. Finally, though we assessed several important time-varying covariates, some covariates that could potentially change during the course of the study including fertility intentions were only assessed at baseline. Though we assume that most women in our analysis, who reported continuing OCP use at each 3-month interval, were not actively trying to get pregnant, we acknowledge that fertility intentions over the three-month interval post-OCP administration may change. This is a limitation of any prospective assessment of unintended pregnancy that is restricted by assessment interval.

## Conclusions

Despite these limitations, our findings clearly indicate the superior efficacy of LARC methods relative to OCPs and condoms alone. We recommend that access to LARC be expanded and that these methods be promoted, particularly among HIV positive and at risk couples wishing to limit or delay fertility. Where transport is affordable and supply chains are reliable, injectables are also an effective pregnancy prevention method. Our work indicates that integration of family planning and HIV counseling and testing services, as well as male partner involvement, is an effective means to achieve this goal that should be further evaluated in non-research settings. Additionally, we recommend that LARC and injectable promotion, and methods to improve OCP adherence, be evaluated to decrease unintended pregnancy outcomes. This study indicates that these interventions could be targeted to younger HIV positive couples that are new OCP users and to those who report missed pills or unprotected sex.
